# Cardiopulmonary Effects of Vagus Nerve Stimulation in Mice

**DOI:** 10.1523/ENEURO.0086-26.2026

**Published:** 2026-09-29

**Authors:** Chris D. Phillips, Christopher M. Driskill, Meena Rajendran, Deepika G. Krishnan, Sven Kroener, Catherine A. Thorn

**Affiliations:** Department of Neuroscience, University of Texas at Dallas, Richardson, Texas 75080

**Keywords:** asymmetry, biomarkers, heart rate, neuromodulation, respiration, vagus nerve

## Abstract

Vagus nerve stimulation (VNS) is an FDA-approved treatment for epilepsy, depression, and chronic stroke. While the effects of VNS are widely studied in rats and large animal models, few studies have characterized VNS in mice due to the practical challenges associated with their small size. However, mouse genetic tools provide a powerful opportunity to elucidate the diverse mechanisms underlying the many effects of VNS. Therefore, to inform the expanding use of mouse models in VNS research, we characterized the cardiopulmonary effects of left versus right VNS in anesthetized C57BL/6 mice. VNS robustly drove decreases in heart rate (HR) and breathing rate (BR) in the majority of mice. VNS-driven changes in BR were not correlated with changes in HR. We found no differences between male and female mice nor in the effects of left versus right VNS. VNS-induced bradycardia was abolished by transection of efferent vagal fibers, consistent with direct descending vagal control of cardiac rhythmicity. In contrast, VNS-driven changes in BR were abolished by both afferent and efferent vagotomies, consistent with the involvement of central feedback circuits in bilaterally coordinated vagal modulation of respiration. These results further our understanding of VNS effects in mice and help inform experimental design and cuff validation protocols for vagal modulation of peripheral versus central circuits in this species.

## Significance Statement

Vagus nerve stimulation (VNS) is under investigation for the treatment of numerous inflammatory and neurological diseases. To optimize VNS therapies, additional research is needed to better understand how VNS exerts its many effects. Mice are an attractive model for mechanistic studies of VNS because of their extensive genetic toolkit, but their small size complicates electrode implantation and validation of stimulation efficacy. Here, we applied VNS in mice to demonstrate that VNS-evoked decreases in heart rate versus breathing rate reflect the independent activation of peripherally versus centrally directed vagal fibers, respectively. These experiments establish a straightforward approach for validating VNS cuff function in mice.

## Introduction

The vagus nerve is the 10th and longest cranial nerve and carries sensorimotor fibers innervating the viscera to provide parasympathetic control of diverse functions such as breathing, heart rate (HR), digestion, and many others (Berthoud and Neuhuber, 2000). Vagus nerve stimulation (VNS) is an FDA-approved therapy used to treat epilepsy (Toffa et al., 2020), depression (Austelle et al., 2022), and chronic stroke (Dawson et al., 2021), and preclinical research is rapidly expanding the potential applications of this therapy to additional neurological disorders and inflammatory diseases (Breit et al., 2018; Aniwattanapong et al., 2022; de Melo et al., 2025). While VNS has been broadly studied in large animal models and in rats, few labs have characterized the effects of VNS specifically in mice, which provide an array of useful genetic tools that could be leveraged to better understand the mechanisms and improve the efficacy of VNS therapies. Due to their small size, mice present a particularly challenging animal model in which to implant VNS cuff electrodes and reliably monitor the physiological effects of stimulation.

In the current study, we aimed to characterize the cardiopulmonary effects of cervical VNS in anesthetized mice, as these effects are commonly used in larger animal models to validate chronic cuff function at the time of implantation. As asymmetric effects of left versus right VNS have been observed in some prior studies (Ardell and Randall, 1986; Cohn, 1912; Robinson and Draper, 1912; Stauss, 2017), we additionally aimed to compare the impact of stimulation side on VNS-driven changes in HR and breathing rate (BR) in mice. Consistent with effects seen in other species, we found that acutely implanted VNS cuff electrodes induced robust reductions in HR, which were abolished following lesions to descending vagal fibers. VNS-driven reductions in BR were also observed in the majority of mice. However, unlike in rats, reductions in BR were not found to be correlated with reductions in HR. VNS-driven changes in BR were abolished by lesions to either ascending or descending vagal fibers, consistent with the engagement of central feedback circuits in bilaterally coordinated vagal modulation of respiration (Song et al., 2015). No significant differences in VNS efficacy were observed between stimulation delivered to the left versus right vagus nerve. Our results provide a novel characterization of VNS effects in mice and demonstrate that, in this species, VNS-driven changes in HR and BR can be used to functionally validate the activation of peripheral versus central pathways, respectively, informing the design of experimental protocols.

## Materials and Methods

All animal procedures were conducted in accordance with the National Institutes of Health Guide for the Care and Use of Laboratory Animals and were approved by the Institutional Animal Care and Use Committee.

### Animal subjects

Male (M: *n* = 34) and female (F: *n* = 28) C57BL/6 mice (https://www.jax.org/strain/000664), aged 10–15 weeks at surgery were used (Table 1). Mice were group housed prior to experiments at 22°C with a 12 h light:12 h dark cycle (ZT0 = 6 A.M.). Food and water was provided *ad libitum*.

**Table 1.** Allocation of mice to experimental groups

Mouse name	HR	SPO_2_	BR	Afferent vagotomy	Efferent vagotomy	BR video + ECG
LVNS1 (F)	X	X				
LVNS2 (M)	X	X				
LVNS3 (F)	X	X				
LVNS4 (F)	X	X				
LVNS5 (M)	X	X	X			
LVNS6 (M)	X	X				
LVNS7 (F)	X	X	X			
LVNS8 (F)	X	X	X			
LVNS9 (M)	X	X	X			
LVNS10 (M)	X	X	X			
LVNS11 (M)	X	X	X			
LVNS12 (M)	X	X	X			
LVNS13 (M)	X	X	X			
LVNS14 (M)	X	X	X			
LVNS15 (M)	X	X	X			
LVNS16 (M)	X	X	X			
LVNS17 (M)	X	X	X			
LVNS20 (F)	X	X	X			
LVNS21 (F)	X	X	X			
LVNS22 (F)	X	X	X			
LVNS24 (F)	X	X	X			
LVNS25 (F)	X	X	X			
LVNS31 (M)						X
LVNS32 (M)						X
RVNS1 (F)	X	X				
RVNS2 (M)	X	X				
RVNS3 (M)	X	X				
RVNS4 (M)	X	X	X			
RVNS5 (F)	X	X				
RVNS6 (F)	X	X	X			
RVNS7 (F)	X	X				
RVNS8 (M)	X	X	X			
RVNS9 (F)	X	X	X			
RVNS10 (M)	X	X	X			
RVNS11 (M)	X	X	X			
RVNS12 (M)	X	X	X			
RVNS13 (M)	X	X	X			
RVNS14 (M)	X	X	X			
RVNS15 (M)	X	X	X			
RVNS16 (M)	X	X	X			
RVNS17 (M)	X	X	X			
RVNS20 (F)	X	X	X			
RVNS21 (F)	X	X	X			
RVNS22 (F)	X	X	X			
RVNS26 (F)	X	X	X			
RVNS27 (F)	X	X	X			
VGAL1 (F)	X	X	X	X		
VGBL2 (M)	X	X	X		X	
VGBL3 (F)	X	X	X		X	
VGAL4 (M)	X	X	X	X		
VGAL5 (F)	X	X	X	X		
VGAL6 (M)	X	X	X	X		
VGBL7 (M)	X	X	X		X	
VGBL8 (F)	X	X	X		X	
VGAR1 (M)	X	X	X	X		
VGBR2 (F)	X	X	X		X	
VGAR3 (F)	X	X	X	X		
VGAR5 (F)	X	X	X	X		
VGAR6 (M)	X	X	X	X		
VGBR8 (F)	X	X	X		X	
VGBR9 (M)	X	X	X		X	
VGBR10 (M)	X	X	X		X	

### VNS cuff construction

Miniaturized mouse VNS cuff electrodes were constructed from a 2.5–3.5 mm length of micro-renathane tubing [BrainTree Scientific, MRE 040320, ID: 0.012″ (0.304 mm), OD: 0.025″ (0.635 mm)], using protocols adapted from those described by Rios et al. (2019) for the manufacture of rat VNS electrodes. Two 3 cm silk sutures were sewn into place on each side of the cuff at the center of the longitudinal axis and secured with UV-curing glue (Loctite, part number, 3106), allowing for flexible manipulation of the cuff during assembly and secure closure of the cuff during surgery. The ends of two 2.5 cm platinum–iridium wires (Sigmund Cohn, part number, 10IR9/49T) were deinsulated, sewn into position inside the cuff, and secured in place with UV-curing glue (Fig. 1*A*,*B*). The platinum–iridium leads were soldered to interface connectors (Omnetics, part number, A24002-004) and tested for proper impedance (0.5–5.0 kΩ at 1 kHz).

### VNS cuff implantation

Mice were anesthetized with isoflurane (induction, 5%; maintenance, 2.5% inhaled), and an incision was made in the skin of the neck to target the left (*n* = 18 M, 14 F) or right (*n* = 16 M, 14 F) vagus nerve at the cervical level. The overlying muscles were then blunt dissected, and the left or right vagus nerve was carefully exposed and dissected from the carotid sheath. The nerve was then gently inserted into the cuff electrode and secured in place with sutures (Fig. 1*C*). Lead wires were connected to an isolated pulse stimulator (A-M Systems, Model 2100).

### Quantification of VNS-driven changes in HR, blood oxygen saturation (SpO_2_), and BR

In all mice, HR and SpO_2_ were continuously monitored under anesthesia using commercially available infrared sensors placed either on the paw (Kent Scientific, Mouse Stat Jr) or on the thigh (Starr Life Sciences, MouseOx Plus). In a subset of mice (*n* = 28 M, 22 F), breathing-related motion of the chest and abdomen was continuously recorded at 30 frames per second using a Logitech C270 video camera (part number, 960-000694). During experiments, 3–5 trials were performed in which a single train of VNS was delivered (train duration, 10 s; pulse amplitude, 0.4 mA; pulse frequency, 30 Hz; total biphasic pulse width, 200 µs; phase width, 100 µs), using an intertrial interval of 60 s.

### Assessment of afferent and efferent regulation of VNS-driven effects

Vagotomies were performed in 16 mice (8 M, 8 F) to determine if the VNS-induced physiological changes were mediated by activation of afferent or efferent vagal fibers. After cuff implantation and experimental procedures, the vagus nerve was cut either rostral [afferent vagotomy; left VNS (L), *n* = 2 M*,* 2 F; right VNS (R), *n* = 2 M*,* 2 F] or caudal (efferent vagotomy; *n* = 2 ML, 2 MR, 2 FL, 2 FR) to the cuff electrode. The nerve and electrode were suspended with suture to avoid contact of the cuff electrode with the adjacent tissue during subsequent stimulation delivery, and the stimulation protocol was repeated. HR, SpO_2_, and BR were monitored continuously before and after vagotomy in all mice included in these experiments.

### Statistical analysis

All analyses of HR, SpO_2_, and BR data were performed in MATLAB. For HR and SpO_2_, measurements were recorded 1 s before VNS delivery (baseline measurement, “BL”) and again at the offset of VNS delivery (post-VNS measurement, “VNS”). For each mouse, the average HR and SpO_2_ were calculated across all trials for BL and VNS time periods. A percent change from BL was computed for each mouse from these average BL and VNS measures. Paired *t* tests were used to determine whether there was a significant change in HR or SpO_2_ between the BL and VNS time points across all subjects. A two-way ANOVA was used to test for significant effects of stimulation side and sex on VNS-evoked changes in HR or SpO_2_. Results are reported as statistically significant for *p* < 0.05.

To calculate BR from each video, average pixel intensity was first computed within a manually selected region of interest (ROI) centered on an area of the chest and/or abdominal wall that exhibited maximum movement. To remove DC and low frequency changes in lighting intensity that were unrelated to breathing movements, the average pixel intensity signal was low-pass filtered by smoothing over a moving 3.3 s window, and this moving average was subtracted from the raw trace. Breaths were easily identifiable from the DC-subtracted traces as brief, high-amplitude, positive- or negative-going deflections from 0. The timing of each breath was defined as the time at which the amplitude of the DC-subtracted trace exceeded a manually determined threshold between 20 and 45% of the maximum (for positive-going breath deflections) or minimum (for negative-going breath deflections) amplitude. For the BL period, BR was computed by averaging the interbreath intervals (IBI) for up to 10 s prior to stimulation if at least 2 breaths were captured (BR = 1/mean IBI). During the 10 s stimulation period (VNS), BR was calculated from IBIs if at least 2 breaths were recorded; otherwise, the number of breaths was divided by 10 to obtain the number of breaths per second. A paired *t* test was used to determine whether there was a significant change in BR between the BL and VNS time points across all subjects. A two-way ANOVA was used to test for significant effects of stimulation side and sex on VNS-evoked changes in BR. Statistical significance is reported for *p* < 0.05.

To validate that BR could be accurately captured from video recordings, in two male mice, we simultaneously recorded video and ECG signals from a pair of stainless steel wire electrodes (A-M Systems, catalog #792400) placed under the skin on either side of the abdomen just below the rib cage. ECG signals were low-pass filtered (0–6 Hz), and five 10 s baseline (no stimulation) periods were extracted from each mouse for comparison of breath times captured by ECG versus video analysis. The timing of each breath was extracted from the videos as described above. Breath times were defined in each 10 s ECG recording as the time at which the absolute value of the low-pass filtered ECG signal exceeded 3 standard deviations from the mean. To compare the ECG and video-based methods, we calculated the Pearson's linear correlation of the detected breath times across all ten 10 s epochs, as well as the absolute value of the difference in breath times between the two methods. Statistical significance is reported for *p* < 0.05.

## Results

### Left and right VNS drive similar reductions in HR in anesthetized mice

We first asked whether left and right VNS drive similar reductions in HR in anesthetized mice, as asymmetry has been reported in other species (Ardell and Randall, 1986; Cohn, 1912; Robinson and Draper, 1912; Stauss, 2017). VNS cuff electrodes were constructed in-house and implanted around the left or right vagus nerves of 60 adult mice (Fig. 1*A*–*C*). Ten-second trains of VNS pulses (amplitude = 0.4 mA, pulse frequency = 30 Hz, total biphasic pulse width = 200 µs) typically drove a decrease in HR that was easily observable using commercially available infrared HR monitors designed for small laboratory animals [Fig. 1*D*; HR (bpm): BL = 453.60 ± 8.74, VNS = 308.05 ± 13.10, mean ± SEM; *p* < 0.0001, paired *t* test]. A two-way ANOVA did not reveal any significant effects of implantation side or sex on these VNS-driven HR reductions (Fig. 1*E*; HR percent change from BL: ML = −31.86 ± 4.11, MR = −25.02 ± 3.52, FL = −38.33 ± 5.34, FR = −35.52 ± 5.70, mean ± SEM; *p*_side_ = 0.3050, *p*_sex_ = 0.0738, *p*_int_ = 0.6669, two-way ANOVA). On average, VNS resulted in a 32.4% decrease in HR. Using a −10% change from BL to define an easily identifiable stimulation-driven decrease in HR that could be used during surgery to validate cuff function, we found that VNS-driven cardiac responses met this threshold in 86% of mice tested (*n* = 52/60).

**Figure 1.**
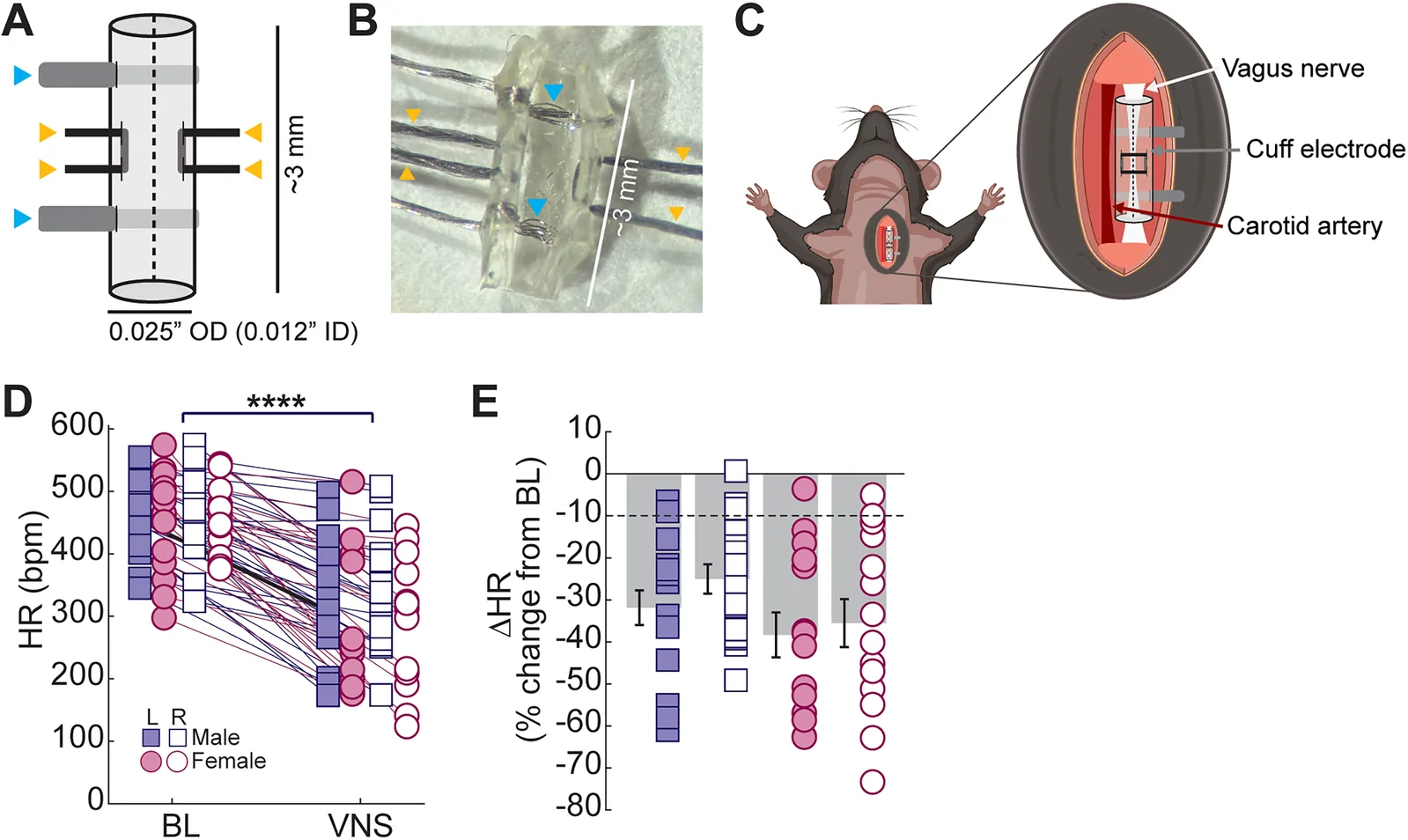
VNS decreases HR in anesthetized mice. ***A***, Diagram illustrating cuff construction of in-house manufactured VNS electrodes for mouse implantation. Micro-renathane tubing is cut open along the longitudinal access (dashed line); two platinum:iridium leads (cyan arrowheads) are sewn into place and secured with UV cure glue; suture is sewn into place on both sides of the cuff (gold arrowheads) at the longitudinal midpoint to facilitate cuff manipulation during assembly and for secure cuff closure during implantation. ***B***, Photograph of assembled mouse VNS cuff electrode (arrowheads denote leads and sutures as in ***A***). ***C***, Cuff electrodes were acutely implanted around the right or left cervical vagus nerve in anesthetized mice. ***D***, VNS (10 s trains of 0.4 mA, 30 Hz pulses) produced a significant decrease in HR; ****p < 0.0001, paired *t* test. ***E***, No significant differences were observed in VNS-evoked HR changes between right and left nerve stimulation or between male and female mice.

VNS also typically evokes a brief cessation of breathing, which, in rats, is accompanied by a significant decrease in SpO_2_ during continuous surgical monitoring (Rios et al., 2019; Sanchez et al., 2020). Although we were able to observe a VNS-driven cessation of breathing in most animals, we found that SpO_2_ was difficult to measure reliably in our mice. The paw sensor often provided low (<70%) readings without other signs of severe hypoxia present and readings were often highly variable from one trial to the next, consistent with poor measurement accuracy. Poor measurement reliability is not unexpected given the difficulty of optimizing optical sensor placement on the small surface area of the mouse paw. Of the 50 mice in which the paw sensor was used to record SpO_2_, 36 (8 ML, 10 MR, 9 FL, 9 FR) had stable baseline measurements above 70%. In these animals, we did not observe a significant VNS-driven change in SpO_2_ [SpO_2_ (%): BL = 84.05 ± 1.18, VNS = 82.95 ± 1.05, mean ± SEM; *p* = 0.1606, paired *t* test]. The thigh sensor was used in 10 female mice and consistently provided stable baseline SpO_2_ readings of >95%. However, this group also failed to exhibit a VNS-driven change in SpO_2_ [SpO_2_ (%): BL = 98.26 ± 0.11, VNS = 97.9 ± 0.26, mean ± SEM; *p* = 0.2229, paired *t* test].

Overall, we find that VNS evokes decreases in HR in anesthetized mice that are easily recorded with commercially available, noninvasive small animal monitors. Moreover, left and right VNS evoked equivalent decreases in HR in both male and female mice. However, we found SpO_2_ measurements to be an unreliable readout of VNS efficacy, consistent with known challenges associated with noninvasive SpO_2_ monitoring in mice.

### Left and right VNS drive similar reductions in BR in anesthetized mice

Because we could not use SpO_2_ measurements to assess VNS-evoked changes in respiratory activity, we used video recordings to capture the effects of VNS on BR in 50 mice. Video recordings provide a simple, low-cost method for monitoring movements of the chest wall related to respiration that remain uncontaminated by electrical artifacts from VNS delivery. To validate the accuracy of video monitoring of BR, we conducted simultaneous ECG and video recordings in two mice (Fig. 2*A*,*B*). Our results indicate that breaths could be detected from the video recordings with high accuracy. During baseline periods of no stimulation delivery, we obtained 100% overlap in detected breaths and excellent alignment of detected breath times between the ECG and video-based methods (Fig. 2*C*; *R*^2^ = 0.9999, *p* < 0.0000, Pearson's linear correlation; difference in breath times: 0.0756 ± 0.0067 ms, mean ± SEM).

**Figure 2.**
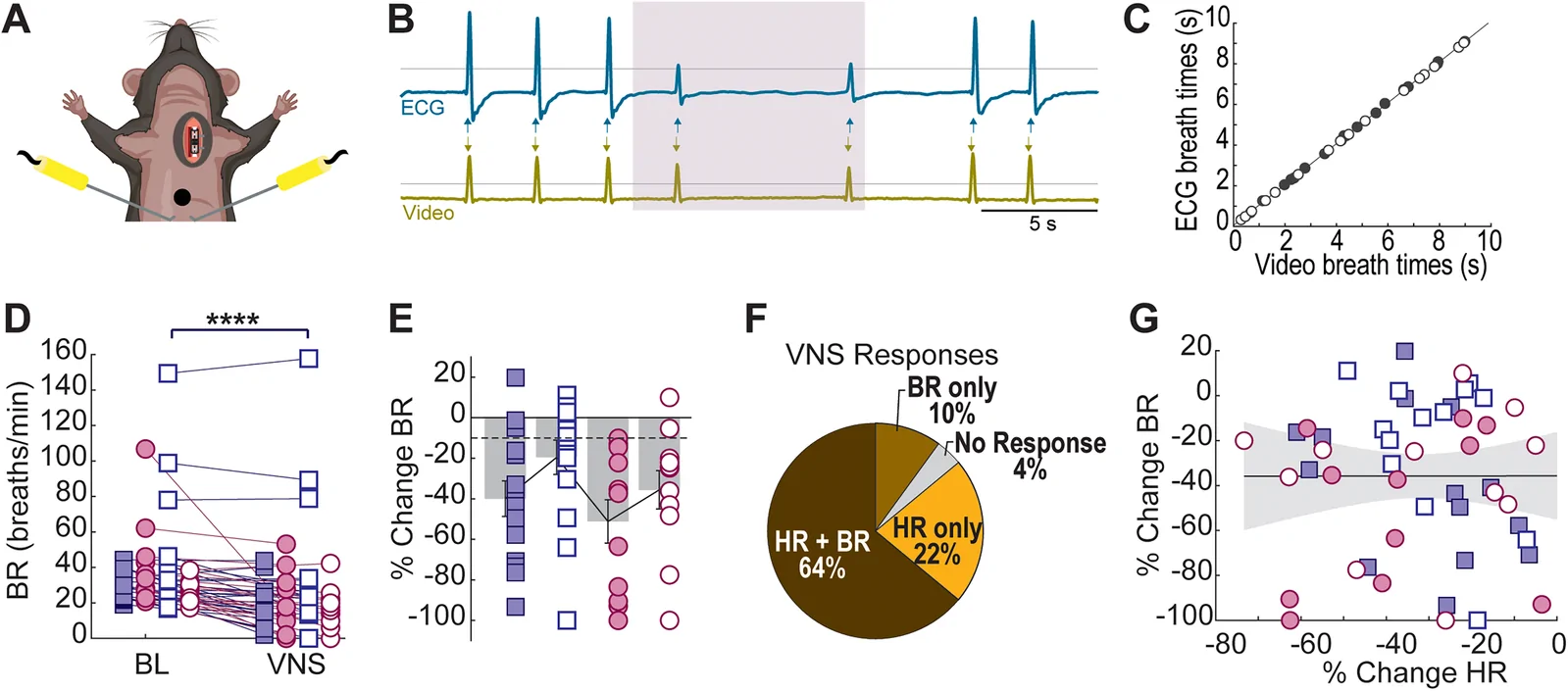
VNS decreases BR in anesthetized mice. ***A***, Video-based breathing analyses were validated in two mice using simultaneously recorded ECG. ***B***, Example breathing signals extracted from ECG (top, cyan) and video (bottom, gold) during a single 30 s recording from one mouse. Gray horizontal lines indicate the thresholds used to detect breaths in each trace, arrows indicate the times of detected breaths in each signal; shaded gray box indicates timing of VNS delivery. ***C***, Across ten 10 s recording epochs, the timing of detected breaths was highly correlated between ECG and video recording methods (*R*^2^ = 0.9999, *p* = 0.0000, Pearson's linear correlation); filled circles, Mouse 1 data; open circles, Mouse 2 data. ***D***, VNS produced a significant decrease in BR; ****p < 0.0001, paired *t* test. ***E***, No significant differences were observed in VNS-evoked BR changes between right and left nerve stimulation or between male and female mice. ***F***, The majority of mice exhibited both HR and BR reductions in response to VNS, though in approximately one-third of animals, only one type of response was observed. ***G***, There was no correlation between the VNS-evoked change in HR and the change in BR. Legend for panels ***D***, ***E***, and ***G*** is shown in Figure 1*D*.

Our experiments confirmed that 10 s trains of VNS drove a decrease in BR, consistent with findings reported in larger species [Fig. 2*D*; BR (breaths/min): BL = 36.12 ± 3.46, VNS = 24.70 ± 3.69, mean ± SEM; *p* < 0.0001, paired *t* test]. A two-way ANOVA did not reveal any significant effects of implantation side or sex on VNS-induced BR changes (Fig. 2*E*; BR percent change from BL: ML = −39.99 ± 1.72, MR = −19.56 ± 5.84, FL = −51.18 ± 2.43, FR = −35.58 ± 1.57, mean ± SEM; *p*_side_ = 0.0600, *p*_sex_ = 0.1520, *p*_int_ = 0.7972, two-way ANOVA). On average, VNS evoked a 35.8% reduction in BR. Using a 10% change from BL to define a decrease in BR that could be easily detected during cuff implant surgery, we found that 74% of mice met this threshold for VNS-driven respiratory effects (*n* = 37/50).

To determine whether HR and BR conveyed similar or independent information related to VNS cuff function, we examined whether there was a relationship between VNS-driven changes in the two parameters. We found that the majority of mice exhibited VNS-driven decreases in both BR and HR (*n* = 32/50, 64%; Fig. 2*F*), and overall failure rates were similar between the two physiological responses (HR: 8/60, BR: 13/50, *p* = 0.0924, *χ*^2^). In a subset of mice, however, VNS drove significant changes to HR (*n* = 11/50, 22%) or BR (*n* = 5/50, 10%), but not both (Fig. 2*F*). There was no correlation between VNS-driven changes in HR and BR across all mice (Fig. 2*G*, *r* = −0.0009, *p* = 0.9948, Pearson's correlation), nor was any relationship observed when only those mice that exhibited significant VNS-driven changes in both parameters were considered (*r* = 0.1072, *p* = 0.5592, Pearson's correlation).

Combined, our data suggest that stimulation-evoked decreases in HR and BR are each robust indicators of VNS cuff function, but these physiological effects arise from independent cardiac and respiratory vagal pathways that may be differentially activated by stimulation in mice.

### VNS-driven HR reductions are driven by efferent vagal fibers

Because of the spatial constraints of VNS in mice, increased variability in VNS-evoked effects may partly result from current leakage and imprecise activation of vagal fibers or adjacent tissue. We therefore validated that VNS-driven changes in HR and BR were attributable to the activation of vagal fibers and assessed the specific contribution afferent versus efferent fibers, by performing nerve transections either rostral or caudal to the level of the cuff, respectively. Transection of the afferent vagal fibers had no impact on VNS-evoked decreases in HR [Fig. 3*A*–*C*; rostral transection, HR (bpm): prevagotomy: BL = 433.40 ± 17.72, VNS = 249.33 ± 26.20, mean ± SEM; *p* < 0.0001, paired *t* test; postvagotomy: BL = 447.95 ± 20.52, VNS = 211.98 ± 28.83, mean ± SEM; *p* < 0.0001, paired *t* test]. In contrast, transection of the efferent vagal fibers abolished the VNS-induced HR changes [Fig. 3*D*–*F*; caudal transection, HR (bpm): prevagotomy: BL = 449.26 ± 32.69, VNS = 274.12 ± 39.41, mean ± SEM, *p* = 0.0035, paired *t* test; postvagotomy: BL = 473.41 ± 27.34, VNS = 472.43 ± 27.07, mean ± SEM, *p* = 0.4985, paired *t* tests]. These data confirm that VNS-driven decreases in HR were mediated by direct activation of efferent vagal fibers.

**Figure 3.**
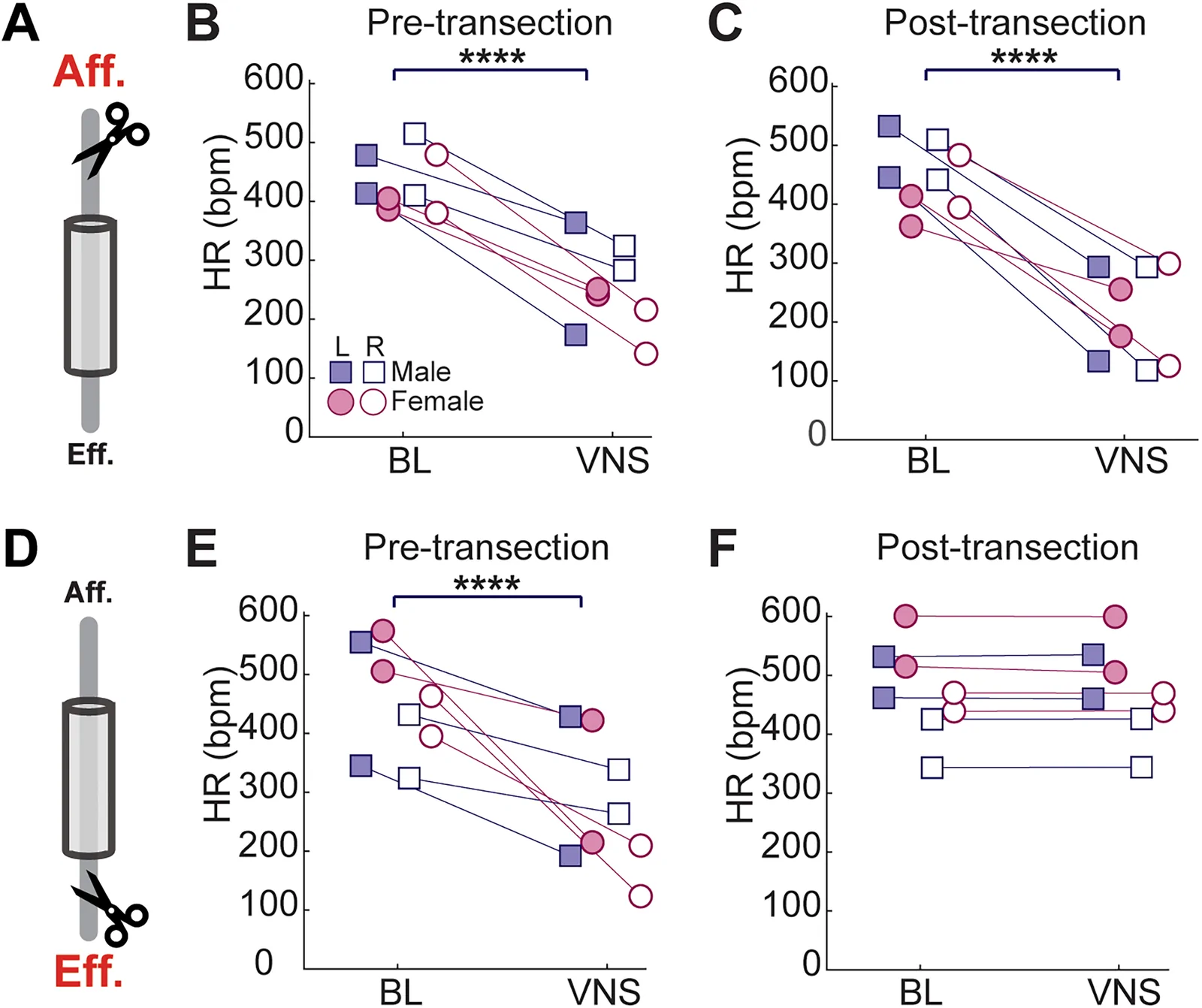
VNS-evoked decreases in HR depend on activation of efferent vagal fibers. ***A***, Transection of afferent (Aff.) vagal fibers was performed in eight mice (*n* = 2 ML, 2 MR, 2 FL, 2 FR). ***B***, Prior to afferent fiber transection, VNS-evoked robust decreases in HR. ***C***, VNS-evoked HR decreases were not altered by transection of afferent vagal fibers. ***D***, Transection of efferent (Eff.) vagal fibers was performed in eight mice (*n* = 2 ML, 2 MR, 2 FL, 2 FR). ***E***, Prior to efferent fiber transection, VNS evoked robust HR reductions. ***F***, After efferent fiber transection, VNS-driven decreases in HR were abolished. ****p < 0.0001, paired *t* test.

### VNS-induced BR reductions require intact afferent and efferent vagal pathways

In the same experiments, we tested whether VNS-driven changes in BR are mediated by afferent or efferent vagal activation. Two mice that did not exhibit VNS-driven decreases in BR (>10% change from BL) prior to the vagotomies were excluded from these analyses (1 MR afferent vagotomy and 1 MR efferent vagotomy). We found that VNS-driven decreases in BR were abolished following transection of afferent vagal fibers [Fig. 4*A*–*C*; rostral transection, BR (breaths/min): prevagotomy: BL = 27.89 ± 3.26, VNS = 16.18 ± 1.87, mean ± SEM; *p* = 0.0022, paired *t* test; postvagotomy: BL = 32.04 ± 4.03, VNS 34.72 ± 6.85, mean ± SEM; *p* = 0.7060, paired *t* test]. VNS-driven changes in BR were also abolished following transection of efferent vagal fibers [Fig. 4*D*–*F*; caudal transection, BR (breaths/min): prevagotomy: BL = 28.43 ± 2.47, VNS = 11.93 ± 4.73, mean ± SEM; *p* = 0.0040, paired *t* test; postvagotomy: BL = 29.61 ± 4.19, VNS = 23.69 ± 3.88, mean ± SEM; *p* = 0.1155, paired *t* test]. These data validate that VNS-driven decreases in BR were due to direct activation of vagal fibers and are consistent with activation of autonomic sensorimotor feedback pathways that require intact afferent and efferent vagal fibers bilaterally.

**Figure 4.**
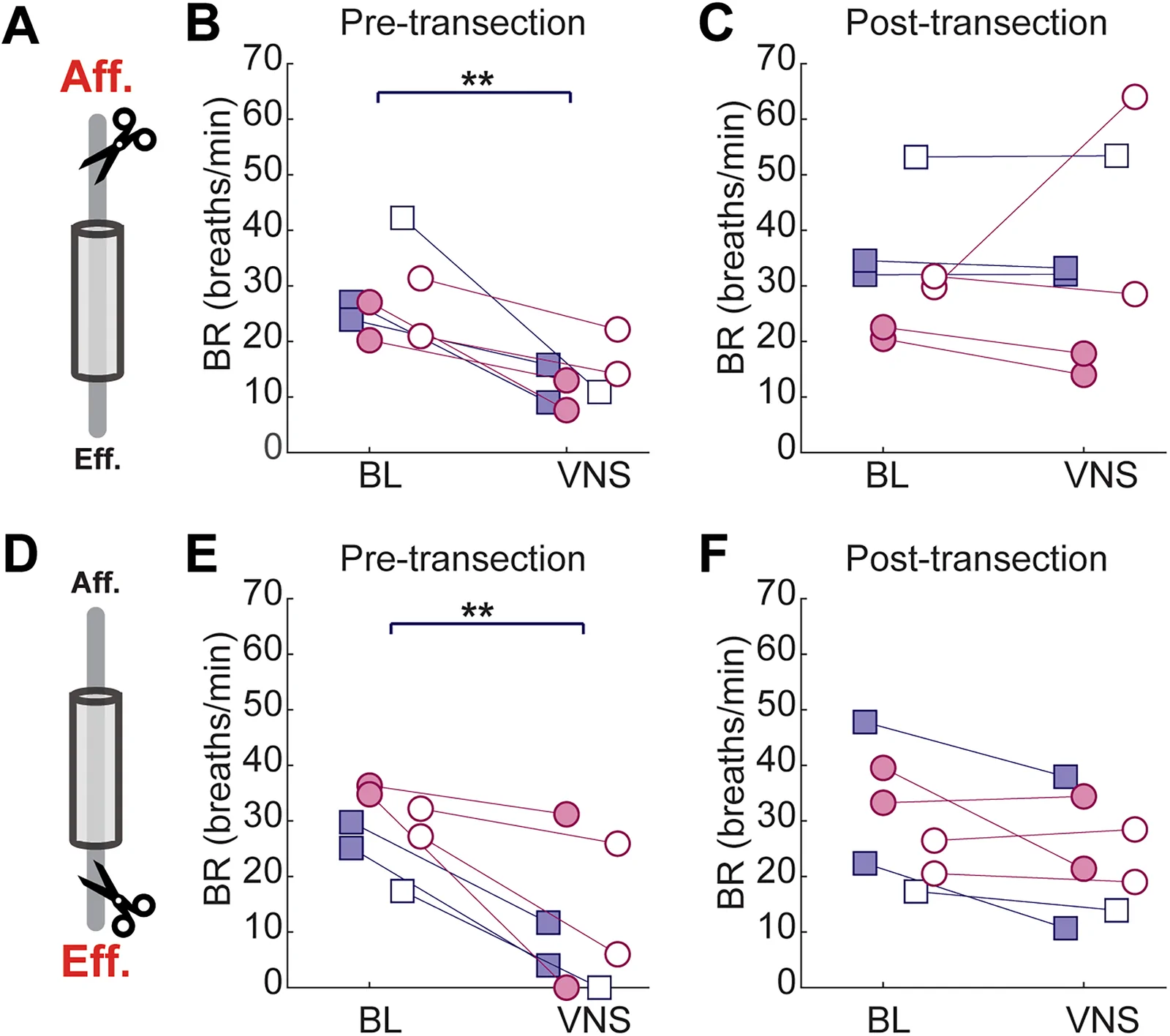
VNS-evoked decreases in BR depend on activation of afferent and efferent vagal fibers. ***A***, Transection of afferent (Aff.) vagal fibers was performed in eight mice (same mice as in Fig. 3*A*–*C*). ***B***, Prior to afferent fiber transection, 7/8 mice (87.5%) exhibited VNS-evoked decreases in BR; **p < 0.01, paired *t* test. ***C***, VNS-evoked decreases in BR were abolished by transection of afferent vagal fibers. ***D***, Transection of efferent (Eff.) vagal fibers was performed in eight mice (same mice as in Fig. 3*D*–*F*). ***E***, Prior to efferent fiber transection, 7/8 mice (87.5%) exhibited significant VNS evoked decreases in BR; **p < 0.01, paired *t* test. ***F***, After efferent fiber transection, VNS-driven decreases in HR were abolished. Legend for panels ***B*** and ***C*** and ***E*** and ***F*** is shown in Figure 3*B*.

## Discussion

The current study provides a novel characterization of cardiopulmonary responses driven by left and right VNS in anesthetized mice to inform the increasing use of this species in mechanistic studies of VNS effects. Consistent with previous reports in mice (Mughrabi et al., 2021; Sun et al., 2023) and other species, VNS evoked significant decreases in HR and BR. Left and right cervical vagal stimulation were equally effective at driving these physiological responses. Interestingly, we find that VNS-evoked changes in HR and BR were uncorrelated with each other, suggesting that independent and fiber-specific activation within the cervical vagus nerve can occur even with the strong, suprathreshold stimulation used here. VNS-driven decreases in HR arise via activation of efferent vagal fibers, whereas decreases in BR required intact afferent and efferent fibers. Our results suggest that these easy-to-monitor physiological responses can be used during cuff implantation or following behavioral assays to validate the successful engagement of peripheral or central neural circuits by VNS in mice.

The sinoatrial node receives asymmetric input from the descending left and right vagus nerves, with greater innervation by right-side fibers biasing the parasympathetic control of HR. Consistent with this anatomical connectivity, the bradycardic effects of right VNS are often reported to be more prominent than those of left VNS (Ardell and Randall, 1986; Cohn, 1912; Zandstra et al., 2021), but other responses have also been observed (Hamlin and Smith, 1968; Stauss, 2017; Yamakawa et al., 2014). Disparate results may be due to differences in experimental protocols, in which anesthesia, species, and stimulation parameters vary widely. In fact, one recent report found that left VNS in rats produced more pronounced decreases in HR than right VNS (Stauss, 2017), particularly at lower stimulation frequencies than we used here. To our knowledge, our study is the first to directly compare the effects of left versus right VNS in mice. Using identical suprathreshold stimulation trains, we find no significant differences in the HR or BR reductions evoked by left versus right VNS in mice. Here, we specifically examined the effects of parameters similar to those found to induce plasticity in the central nervous system, both in preclinical studies in rats (Porter et al., 2012; Khodaparast et al., 2013) and in clinical studies in stroke patients (Dawson et al., 2021). This focus on a single parameter set, however, represents an important limitation of the current study, as we did not test for differences in activation thresholds between the right and left vagus nerves. Additional research is needed to fully characterize the extent to which stimulation of the left versus right nerves drive differential cardiopulmonary responses across a broader parameter space.

We found that VNS-evoked decreases in HR and BR were independently driven by activation of efferent versus afferent fibers, respectively. Efferent cardiac vagal fibers include rapidly conducting myelinated axons that form fast cholinergic synapses onto cardiac postganglionic neurons to regulate HR (Nosaka et al., 1982; Taylor et al., 1999). In contrast, mixed fiber types ascend to the NTS to modulate brainstem respiratory nuclei through a multisynaptic reflex pathway (Taylor et al., 1999). Independent activation of these anatomically distinct vagal pathways raises the possibility that selective activation may be possible through optimization of stimulation parameters. Whether parameters other than the ones tested here can more selectively activate efferent versus afferent fibers in mice remains an open and important question for future studies.

An additional limitation to the present study is that all recordings were performed under isoflurane anesthesia, which is known to alter autonomic tone and influence the magnitude of cardiopulmonary reflexes elicited by VNS (Skovsted and Sapthavichaikul, 1977; Ahmed et al., 2021). Thus, while these physiological responses serve as a useful biomarker of VNS efficacy and relative engagement of afferent versus efferent fibers during device implantation, they are unlikely to fully predict VNS-driven physiological responses in behaving animals. Ahmed et al. (2021) showed that significantly higher VNS amplitudes are required to drive changes in HR in rats under isofluorane anesthesia, compared with those required during awake or ketamine-anesthetized states. While differences in nerve diameter and composition between rats and mice make it difficult to directly compare data across studies, the results of Ahmed et al. suggest that the 0.4 mA intensity used in the current study is likely well above the threshold required to induce HR changes across states. Interestingly, their data also indicate that, in awake animals, effective VNS intensities are likely to drive significant decreases in HR. As HR is rarely monitored in preclinical studies of VNS, additional work in awake animals would provide important insight into the potential cardiopulmonary side effects of VNS across the range of stimulation patterns used to target peripheral versus neurological indications.

VNS is under investigation as a therapeutic intervention for an increasing number of clinical disorders, heightening the need for mechanistic studies that can inform the optimization of stimulation strategies that best engage targeted cellular pathways. Mouse models enable the use of powerful genetic and experimental techniques, which can be uniquely valuable for dissecting the central and peripheral effects of VNS. As the field moves toward more precise and mechanistically informed applications of VNS, validating the efficacy of VNS in mice will be essential for translating basic discoveries into therapeutic advances. Here, we show that classic VNS-driven decreases in HR and BR are easily observed following stimulation of the left or right cervical vagus nerve in mice. However, HR and BR responses arise from independent activation of efferent versus afferent vagal fibers, respectively, and these pathways may be differentially engaged even by suprathreshold stimulation. These findings are expected to inform the optimization of experimental VNS protocols in mice and highlight the importance of validating VNS efficacy using appropriate circuit-specific biomarkers.

## Synthesis

Reviewing Editor: Gergely Silasi, University of Ottawa Decisions are customarily a result of the Reviewing Editor and the peer reviewers coming together and discussing their recommendations until a consensus is reached. When revisions are invited, a fact-based synthesis statement explaining their decision and outlining what is needed to prepare a revision will be listed below. The following reviewer(s) agreed to reveal their identity: NONE.

Pease find below the detailed comments from an expert reviewer.

Specifically, points 1 and 2 may be addressed by acknowledging these limitations within the discussion. For point 3, would it be possible to confirm the accuracy of the video-based respiration measurements?

Reviewer Comments:

This is an interesting manuscript by Chris D. Phillips et al. that investigated the cardiopulmonary effects of left versus right Vagus Nerve Stimulation (VNS) in anesthetized mice. Using a custom-designed miniature VNS cuff electrode and surgically performed vagotomy, the research team dissected the peripheral and central pathways that mediate bradycardia and respiratory depression induced by VNS. It was revealed that the reduction in heart rate caused by vagus nerve stimulation is mediated by direct descending efferent fibers, whereas the slowing of respiratory rate requires both afferent and efferent pathways to remain intact. This finding provides a robust validation method for mouse models of VNS. This study has broad reference value for researchers in the fields of neuromodulation, autonomic control, and bioelectronic medicine. Below are the major and minor comments for revising the manuscript. Therefore, major revision has to be done before this manuscript could be accepted for publication in the eNeuro.

Major comments:

1. All experiments in this study were performed under deep isoflurane anesthesia. Isoflurane is a potent cardiovascular and respiratory depressant that can significantly alter autonomic tone, which complicates the interpretation of cardiopulmonary reflexes elicited by VNS. The authors must address how this anesthetic condition limits their findings.

2. The authors suggest that left and right VNS produce no differential physiological effects in mice. However, this conclusion is based on a single fixed stimulation paradigm and lacks a parametric scan of stimulation parameters. The authors must provide input-output curves to identify potential differences in activation thresholds.

3. The method used to measure respiratory rate is relatively crude and highly susceptible to motion artifacts. The authors must validate the sensitivity and accuracy of their video-tracking algorithm to meet gold-standard physiological measurement criteria.

Minor comments:

1. The manuscript states that the reduction in BR was not associated with significant changes in SpO₂. However, the Results section acknowledges that this is largely due to poor measurement reliability and frequent sensor failure caused by the small surface area of the mice's extremities. Conclusions regarding SpO₂ must be weakened.

2. In the manuscript, the authors use the terms "above cuff" and "below cuff" to describe afferent and efferent transections. It is recommended that these terms be replaced to avoid reader confusion and to conform to standard anatomical nomenclature.

3. In the manuscript, inappropriate punctuation appears in the section heading "DISCUSSION: ". A full check of the entire text is recommended.

## Author Response

Synthesis of Reviews:

Synthesis Statement for Author (Required):

Pease find below the detailed comments from an expert reviewer.

Specifically, points 1 and 2 may be addressed by acknowledging these limitations within the discussion. For point 3, would it be possible to confirm the accuracy of the video-based respiration measurements? We thank the Editor and Reviewer for their careful consideration of our manuscript and have included additional discussion of the study's limitations and validation of the video analysis of breathing rate, as requested. Below please find a point-by-point response detailing how each of the Reviewer's concerns have been addressed in the revision.

Reviewer 1:

This is an interesting manuscript by Chris D. Phillips et al. that investigated the cardiopulmonary effects of left versus right Vagus Nerve Stimulation (VNS) in anesthetized mice. Using a custom-designed miniature VNS cuff electrode and surgically performed vagotomy, the research team dissected the peripheral and central pathways that mediate bradycardia and respiratory depression induced by VNS. It was revealed that the reduction in heart rate caused by vagus nerve stimulation is mediated by direct descending efferent fibers, whereas the slowing of respiratory rate requires both afferent and efferent pathways to remain intact. This finding provides a robust validation method for mouse models of VNS. This study has broad reference value for researchers in the fields of neuromodulation, autonomic control, and bioelectronic medicine. Below are the major and minor comments for revising the manuscript. Therefore, major revision has to be done before this manuscript could be accepted for publication in the eNeuro.

Major comments:

1. All experiments in this study were performed under deep isoflurane anesthesia. Isoflurane is a potent cardiovascular and respiratory depressant that can significantly alter autonomic tone, which complicates the interpretation of cardiopulmonary reflexes elicited by VNS. The authors must address how this anesthetic condition limits their findings.

To address this concern, we have edited the Discussion to include consideration of this limitation (p 17-18).

2. The authors suggest that left and right VNS produce no differential physiological effects in mice. However, this conclusion is based on a single fixed stimulation paradigm and lacks a parametric scan of stimulation parameters. The authors must provide input-output curves to identify potential differences in activation thresholds.

To address this concern, we have edited the Discussion (p. 17) to explicitly highlight this important limitation and the need for additional studies to fully characterize cardiopulmonary responses across a broader parameter space, including defining activation thresholds for the right vs. left sides.

3. The method used to measure respiratory rate is relatively crude and highly susceptible to motion artifacts. The authors must validate the sensitivity and accuracy of their video-tracking algorithm to meet gold-standard physiological measurement criteria.

We have performed additional experiments to validate the sensitivity and accuracy of the video monitoring method compared to ECG recording, and find excellent alignment in breaths detected between the two methods. The Methods and Results have been edited to incorporate the validation experiments, Figure 2 has been updated to illustrate the results Minor comments:

1. The manuscript states that the reduction in BR was not associated with significant changes in SpO₂. However, the Results section acknowledges that this is largely due to poor measurement reliability and frequent sensor failure caused by the small surface area of the mice's extremities. Conclusions regarding SpO₂ must be weakened.

We have edited the Abstract and Introduction to remove conclusions related to SpO2.

2. In the manuscript, the authors use the terms "above cuff" and "below cuff" to describe afferent and efferent transections. It is recommended that these terms be replaced to avoid reader confusion and to conform to standard anatomical nomenclature.

We have edited the manuscript, replacing "above cuff" and "below cuff" with "rostral" and "caudal" to the level of the cuff, respectively.

3. In the manuscript, inappropriate punctuation appears in the section heading "DISCUSSION: ". A full check of the entire text is recommended.

We have carefully reviewed the manuscript to correct these errors.
